# Retinal Angiomatous Proliferation in a Patient with Retinitis Pigmentosa

**DOI:** 10.3390/genes14071438

**Published:** 2023-07-13

**Authors:** Peter Kiraly, Susan M. Downes, M. Dominik Fischer

**Affiliations:** 1 Oxford Eye Hospital, Oxford University Hospitals NHS Foundation Trust, Oxford OX3 9DU, UK; peter.kiraly20@gmail.com (P.K.);; 2 Nuffield Department of Clinical Neuroscience, University of Oxford, Oxford OX3 9DU, UK; 3 Centre for Ophthalmology, University Hospital Tübingen, 72076 Tübingen, Germany

**Keywords:** retinal angiomatous proliferation (RAP), retinitis pigmentosa (RP), choroidal neovascularization (CNV), inherited retinal dystrophies (IRD)

## Abstract

Retinal angiomatous proliferation (RAP) and other types of choroidal neovascularization (CNV) are very rarely reported in patients with retinitis pigmentosa (RP). We present a case report of a 91-year-old patient with an obvious RP phenotype, who presented with a sudden onset of vision worsening and metamorphopsia in the left eye. Genetic testing on the UK inherited retinal disease panel did not identify a pathogenic variant. Multimodal imaging comprising optical coherence tomography (OCT), OCT angiography, and fluorescein and indocyanine green angiography showed a RAP lesion in the left macula. The patient received three treatments of monthly injections of aflibercept, with excellent morphological and functional outcomes. Taking into account the patient’s age at presentation of the RAP lesion, it is not clear whether the RAP was associated or coincidental with RP. This case report highlights the importance of possessing an awareness that RAP lesions can occur in RP. Moreover, due to a good response and potential safety concerns with continuous anti-VEGF injections in RP patients, a pro re nata (PRN) regimen might be the safest option.

## 1. Introduction

Retinitis pigmentosa (RP) is an inherited retinal dystrophy (IRD) with a heterogenous genotype and phenotype and a prevalence of 1:3000 to 1:7000 [1]. It is characterized by the progressive dysfunction of rod photoreceptors, predominantly, and the subsequent degeneration of cone photoreceptors. Cells of the retinal pigment epithelium (RPE) form characteristic bone spicules in areas with photoreceptor loss [2]. Further phenotypic hallmarks of RP include arteriolar attenuation and a waxy pallor of the optic nerve disc [3]. Most RP patients first notice night blindness, followed by peripheral visual loss under well-lit conditions and severe visual impairment or blindness in the end stage [3]. RP has been associated with posterior subcapsular cataracts, myopia, astigmatism [4], and macular changes, including cystoid macular oedema (CMO) (20.4%) and epiretinal membrane formation (15.6%) [5].

Retinal angiomatous proliferation (RAP) is a type 3 macular neovascularization (MNV) [6]. It is most commonly associated with neovascular age-related macular degeneration (AMD) and accounts for approximately 10–15% of patients with neovascular AMD [7,8]. Neovascularization in RAP originates from the retina and progresses posteriorly to the choroid to form a retinal-choroidal anastomosis [8]. A meta-analysis showed that RAP lesions respond better to anti-VEGF treatment than other neovascular AMD subtypes [9]. Choroidal neovascularization (CNV) has been associated with several IRDs: Stargardt disease, Best vitelliform dystrophy, Sjögren reticular dystrophy, pattern dystrophy, gyrate atrophy, Sorsby fundus dystrophy, and RP [10,11]. In RP, CNV is extremely rare [11], with the majority of case reports describing either classic CNV [12,13,14,15], pachychoroid neovasculopathy [12], or RAP lesions [16,17,18]. In our study, we present a case report of a patient with RP and a RAP lesion diagnosed using multimodal imaging. This is the first case report of a RAP lesion in RP that was treated with intravitreal aflibercept, demonstrating an excellent treatment response.

## 2. Materials and Methods

### 2.1. Ophthalmic Examination

The patient underwent a full clinical examination at the Oxford Eye Hospital, with clinical and genetic testing performed as per the standard of care. All data were collected retrospectively. Since 2016, the patient has been reviewed every two years to monitor their RP. In 2023, the patient presented with a sudden onset of distorted vision. Multimodal imaging was performed, including spectral-domain optical coherence tomography (OCT), OCT angiography, blue fundus autofluorescence (FAF), fluorescein (FFA) and indocyanine green angiography (ICGA) (Spectralis, Heidelberg Engineering, Inc., Heidelberg, Germany), and pseudo-colour Optos (Optomap P200; Optos plc, Dunfermline, UK).

### 2.2. Genetic Testing

Informed consent was obtained for genetic testing at the Oxford Regional Genetics Laboratories. In 2016, molecular genetic analysis for RP and RP-like phenotypes that included an RP 111 Gene Panel was performed. A HaloPlex Target enrichment system (Agilent Technologies, Didcot, UK) was used for the amplification of the coding regions, and Illumina MiSeq (Illumina, San Diego, CA, USA) was used for sequencing. As the gene panel did not detect a pathogenic variant in our patient, she was enrolled in the 100,000 Genomes Project. Whole-genome sequencing was performed by Genomics England, and no underlying genetic cause of the patient’s clinical presentation has been identified thus far.

## 3. Results

The patient was first diagnosed with RP at age 18. Her paternal uncle had poor vision with unknown aetiology. In 2014, the patient underwent a right vitrectomy to release vitreomacular traction and a peel of an epiretinal membrane. The persistent mild vitreomacular traction in the left eye has remained stable for several years. Molecular genetic analysis that included an RP 111 Gene Panel and whole-genome sequencing has not identified a sequence variant to date. In addition to her RP, the patient’s medical history includes past giant cell arteritis, osteoporosis, nodal osteoarthritis, and cardiomyopathy. At the last follow-up, the patient was taking methotrexate, folic acid 5 mg once a week, denosumab injections every six months, calcium carbonate (1500 mg) and vitamin D3 (400 iu) in two tablets daily, atorvastatin 10 mg at night, clopidogrel 75 mg once a day, nitrofurantoin 50 mg once at night, and omeprazole 20 mg once a day.

**Figure 1:**
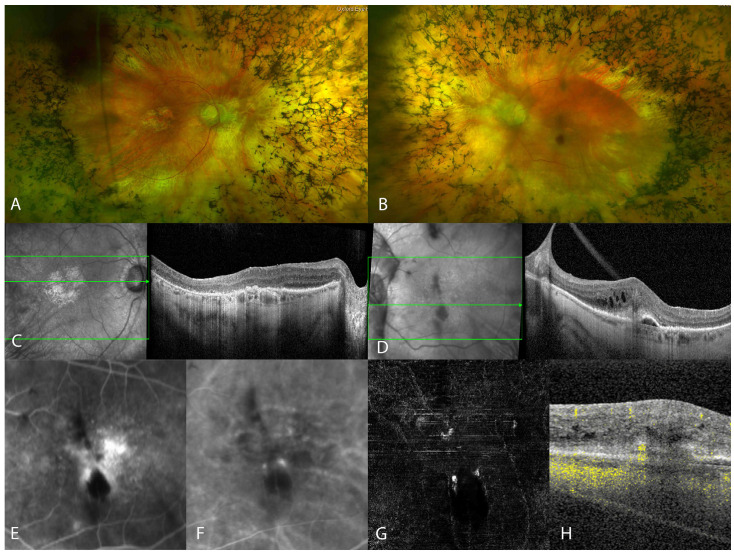
Pseudo-colour images showed optic nerve disc pallor, vascular attenuation, and extensive peripheral bone spicules in the right eye (**A**) and in the left eye (**B**), with an intraretinal haemorrhage just inferior to the fovea. Optical coherence tomography (OCT) in the right eye (**C**) showed irregularities in the outer retinal layers, while OCT in the left eye (**D**) showed intraretinal fluid, subretinal hyper-reflective material, the ‘kissing sign’ between the inner retinal layers and the retinal pigment epithelium, and PED with hyper-reflective and hyporeflective components. Fluorescein angiography (**E**) showed diffuse leakage in the later stages at the fovea, while indocyanine green angiography (**F**) showed hotspot in the mid- to late stages. OCT angiography (**G**,**H**) showed a claw-like lesion and pathological blood flow.

At the age of 91, she presented with sudden vision loss and metamorphopsia in the left eye. Her best-corrected visual acuity (BCVA) in both eyes was 6/24. Intraocular pressure (IOP) was within the normal limits bilaterally. Slit lamp examination revealed quiet pseudophakia in both eyes, with the IOL subluxated inferiorly in the left eye. A fundus examination of both eyes revealed a pale optic nerve disc, vascular attenuation, and extensive peripheral bone spicule pigmentation (Figure 1A,B). Macular pigmentary changes were seen in both eyes, with foveal involving geographic atrophy in the right eye (Figure 1A) and an intraretinal haemorrhage inferior to the fovea in the left eye (Figure 1B). OCT imaging of the right macula showed irregular outer retinal layers with an irregular/disrupted ellipsoid zone (EZ) line and RPE in the fovea (Figure 1C). OCT imaging of the left macula showed persistent stable vitreomacular traction, extensive intraretinal fluid accumulation, numerous hyper-reflective foci, subretinal hyper-reflective material, the ‘kissing sign’ between the inner retinal layers and the retinal pigment epithelium, and PED with hyper-reflective and hyporeflective components (Figure 1D). FFA showed a focal pinpoint hyperfluorescence (hot spot) in the early phase with diffuse leakage in the later phase of angiography in the left macula (Figure 1E). ICGA showed a hotspot in mid- to late-phase images (Figure 1F) corresponding to the topographical area of pinpoint hyperfluorescence, as seen on FFA. On the en face OCTA avascular slab image, a claw-like lesion was seen, and flow was noticed in the previously described ‘kissing sign’, as seen on OCT (Figure 1G,H).

**Figure 2:**
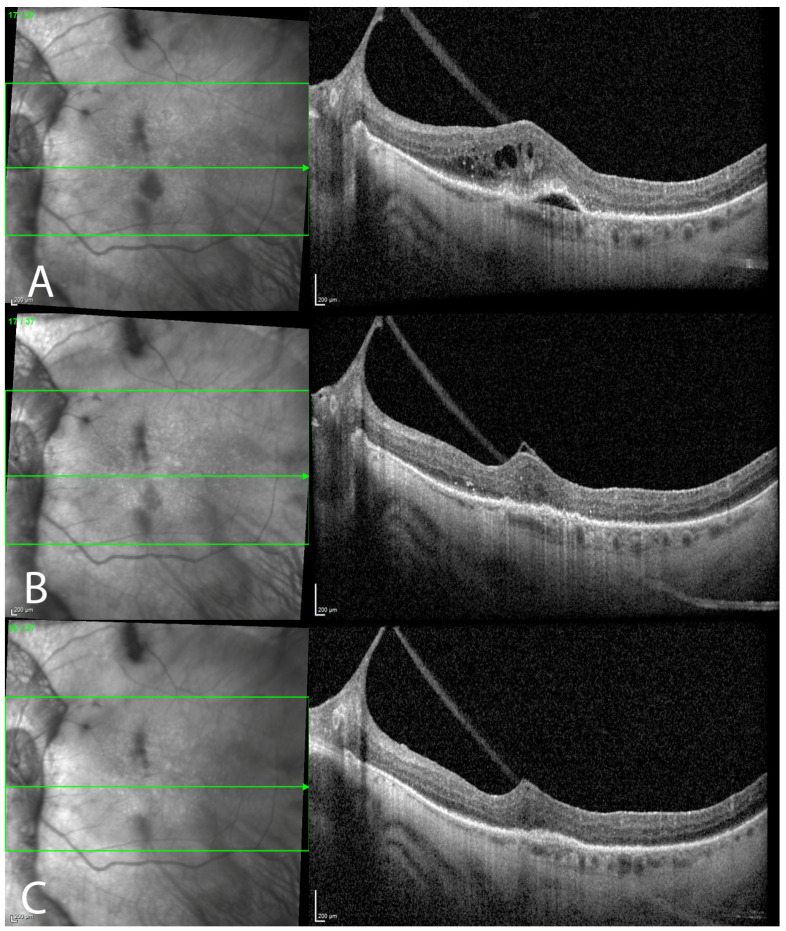
Optical coherence tomography (OCT) prior to treatment shows significant neovascular activity (**A**), with a complete resolution of the intraretinal fluid and subretinal hyper-reflective material, as well as PED flattening, two weeks after a single aflibercept injection (**B**), with no neovascular activity four weeks after three treatments of monthly aflibercept injections (**C**).

After multimodal imaging confirmed the presence of a RAP lesion in the left macula, intravitreal treatment with aflibercept was offered to the patient. Two weeks after a single intravitreal injection, her vision improved from 6/24 to 6/15 in the left eye with a significant subjective vision improvement. OCT of the left macula showed complete resolution of the intraretinal fluid, significant reduction in the hyper-reflective foci, the resolution of the subretinal hyper-reflective material, and flattening of the PED. Four weeks after three treatments of monthly aflibercept injections (loading dose), there were no signs of neovascular activity (Figure 2).

## 4. Discussion

In our report, we describe a case of a unilateral RAP lesion in a patient with RP who showed an excellent functional and morphological response after treatment with intravitreal injections of aflibercept. RP is characterized by the progressive constriction of the peripheral visual field, which can lead to tunnel vision and blindness [1]. Central vision can also be affected earlier due to the CMO, vitreomacular traction, epiretinal membrane, and macular hole [5,19,20]. One of the causes of central vision loss in RP could be a RAP lesion, which is extremely rare, having been reported hitherto in only three case reports [16,17,18].

It has been hypothesized that the IRD-related degeneration of photoreceptors, RPE, and choriocapillaris in RP patients can lead to the formation of a CNV [11,13]. Moreover, retinal degeneration, as seen in RP, can lead to RP-associated CMO, which is hypothesized to be associated with the breakdown of the blood–retinal barrier and alterations in inflammatory cytokines [21]. Although there are no studies assessing the vitreous levels of VEGF in patients with CMO-associated RP [21], several studies showed morphological and/or functional improvement after the intravitreal application of anti-VEGF [22,23,24,25]. In neovascular AMD, VEGF is a key mediator of CNV development, promoting angiogenesis and leading to increased vascular permeability [26]. The formation of idiopathic or secondary epiretinal membranes is thought to be caused by the presence of glial cells, fibroblasts, and hyalocytes within the epiretinal membrane, along with cytokines and growth factors found in the vitreous membrane [27,28,29]. Moreover, according to a study conducted by Mandelcorn et al., VEGF staining was observed in 11 out of 13 idiopathic epiretinal membranes [30]. Our patient developed an epiretinal membrane and vitreomacular traction in both eyes and underwent a vitrectomy and epiretinal membrane peel in the right eye. It is therefore possible that our patient had an upregulation of inflammatory cytokines and VEGF, which could have contributed to the development of a RAP lesion.

CMO can be seen on angiography whether developed in association with RP or as a consequence of a RAP lesion [5,7]. Among RAP lesions, subretinal fluid is present in only 66.7%, and PED is present in 26.2% of cases [7]. It is very important to search for a hot spot on angiography and the presence of intra-retinal haemorrhage, which are present in more than 90% of RAP lesions when distinguishing between RAP in RP and CMO associated with RP [7].

We were unable to determine whether the RAP lesion in our patient was associated with her RP or whether it occurred within the neovascular AMD spectrum independent of the RP. In all case reports where RAP and RP were described, patients were 60 years of age or older [16,17,18]. By contrast, in the case reports with RP and type 2 CNV, the patients were 40 years old [13,31]. In other IRDs, where CNV is an established complication (Sorsby Fundus Dystrophy, Best vitelliform macular dystrophy), patients developed type 2 CNV at a younger age [32,33]. Therefore, this could imply that our patient developed RAP in the spectrum of neovascular AMD.

Alternatively, it may be the case that patients with RP who develop RAP lesions may have a genetic risk factor yet to be identified. In only one case report with RAP in RP, molecular genetic analysis showed variants of uncertain significance within two genes associated with RP [18]. RP exhibits significant phenotypic diversity, with variations in the age of onset and disease progression observed among patients, including within families [34]. Additionally, RP is characterized by a high degree of genetic heterogeneity, with more than 90 genes that are associated with the disorder identified thus far [35]. Initially, a targeted next-generation sequencing (NGS) analysis focusing on 111 genes associated with RP and RP-like phenotypes failed to identify the underlying genetic cause in our patient. The overall success rate of molecular diagnostics using targeted sequencing for RP is generally reported to be around 55–65% [34]. The capability of targeted sequencing is limited by its ability to comprehensively detect genetic variations, such as the identification of extensive genomic deletions or duplications that encompass protein-coding regions, as well as pathogenic variants in the non-coding regions of genes [36]. As a result, disease-causing variants in non-coding regions may remain undetected when using conventional targeted techniques [37]. Moreover, targeted sequencing may fail to detect mutations occurring in genes that have not yet been associated with IRD [37]. In an attempt to identify the disease-causing variant in our patient, she was included in the 100,000 Genomes Project and underwent whole-genome sequencing (WGS). However, even with WGS, the pathogenic variant in our patient could not be identified. WGS enables the detection of all types of variants across the entire human genome, including the identification of deep-intronic variants and structural variants [34]. While the sensitivity of WGS in identifying pathogenic variants in RP has not yet been established, one study showed a significant uplift in diagnostic yield (29%) with WGS, in addition to a 55–65% diagnostic yield reported in panel sequencing [34,36]. Despite the extensive research conducted on exons, our knowledge regarding the non-coding regions remains limited. WGS has revealed additional variants in the non-coding regions, but their classification as pathogenic is often challenging due to a lack of sufficient evidence. In many cases, the interpretation of non-coding variants relies solely on in silico predictions, leading to confusion among clinical geneticists when identifying pathogenic variants. In other words, even with the complete sequence of the genome, there is still inadequate evidence to classify or interpret the detected variants [34].

Our patient responded with a complete resolution of neovascular activity seen at two weeks after a single intravitreal injection of 2.0 mg/0.05 mL aflibercept. Our patient received three treatments of monthly aflibercept injections (loading dose), with no recurrence of neovascular activity four weeks after the last injection. In two other case reports with RAP and RP, the patients were treated with a treat-and-extend protocol and received 8 intravitreal injections of ranibizumab [16] or 10 injections of bevacizumab [18], respectively. One RP patient with classic CNV reportedly needed five intravitreal injections of bevacizumab to stabilize the CNV [15]. The study revealed that eyes with RAP had an 11-fold higher chance of achieving extended remission on a pro re nata (PRN) protocol compared to eyes with type 2 CNV [36]. Moreover, a prospective study showed that eyes with RAP lesions were less frequently associated with fluid at 1 and 2 years during the anti-VEGF therapy in comparison with other CNV types [7].

Knocking out VEGF-A in a mouse model resulted in choriocapillaris atrophy followed by the impairment of photoreceptor function and subsequent vision loss [37]. Large prospective studies showed an increased risk of geographic atrophy in patients treated with monthly anti-VEGF injections in comparison with patients who were treated with the PRN protocol [38,39]. Parodi et al. hypothesized that repeated intravitreal anti-VEGF injections might be unsafe for patients with RP, whose photoreceptors are already compromised [15]. They argued that continued intraocular VEGF neutralization could contribute to reduced retinal and choroidal circulation, which are important neurotrophic factors [15]. Thus, a PRN regimen may be a better treatment protocol option for patients with RAP and RP.

In conclusion, it is important to be aware that RAP lesions can occur in patients with RP. It is not known whether our patient has an unknown RP variant(s) contributing to the phenotype of RAP in RP, or whether this case simply reflects two independent disease entities that coincide in our patient. Intravitreal treatment with aflibercept resulted in a very good morphological and functional outcome in our patient. A PRN approach might be a preferred treatment option due to safety concerns regarding RP patients.

## Data Availability

Not applicable.
